# Preclinical toxicological assessment of levothyroxine and liothyronine Maillard impurities

**DOI:** 10.1093/toxres/tfac047

**Published:** 2022-08-13

**Authors:** Anju Agarwal, Muhammad Asif, Rajeev Deshmukh, Mohini Vinchurkar, Suresh B Singana, Pratik Bhondave

**Affiliations:** ADVANZ PHARMA, Capital House, 85 King William Street, London, EC4N 7BL, United Kingdom; ADVANZ PHARMA, Capital House, 85 King William Street, London, EC4N 7BL, United Kingdom; ADVANZ PHARMA, Capital House, 85 King William Street, London, EC4N 7BL, United Kingdom; ADVANZ PHARMA, Capital House, 85 King William Street, London, EC4N 7BL, United Kingdom; ADVANZ PHARMA, Capital House, 85 King William Street, London, EC4N 7BL, United Kingdom; ADVANZ PHARMA, Capital House, 85 King William Street, London, EC4N 7BL, United Kingdom

**Keywords:** preclinical, levothyroxine and liothyronine, Maillard, impurities, toxicological assessments

## Abstract

**Background:**

Following the introduction of new stability-indicating related substances methods, an unknown impurity was observed in levothyroxine (LeMI) and liothyronine (LiMI) tablets (ADVANZ PHARMA) in concentrations ≥1.0%, from 6 months of storage onwards. The impurity was identified as a Maillard condensation product between lactose and LeMI/LiMI in the LeMI and LiMI tablets, respectively.

**Materials and Methods:**

To establish the toxicity profile of LeMI and LiMI in humans and to define appropriate shelf-life specification limits, a comprehensive nonclinical toxicological assessment was performed, including *in silico* (Leadscope and Derek Nexus analyses), *in vitro* (Ames test), and *in vivo* tests (7-day dose range finding and 90-day dose repeat studies in rats). *In silico* analyses indicated that potential LeMI and LiMI structures should not be considered bacterial mutagens or *in vitro*/*in vivo* clastogens, and that at the low oral exposure levels expected, the impurities are unlikely to cause harm.

**Results:**

*In vitro* testing showed that neither LeMI nor LiMI were cytotoxic or mutagenic at up to 5000 μg/plate, both in the presence and absence of metabolic activation. The 2 *in vivo* studies further confirmed that no systemic toxicity or other notable negative effects were evident at up to 200 μg/kg/day for LeMI and 45 μg/kg/day for LiMI, the highest doses tested. These doses represent 120–122 times the maximum daily exposures of LeMI and LiMI, based on body surface area (μg/m^2^).

**Conclusions:**

Based on these results, a proposal has been formulated to increase the limits of Maillard condensation products to ≤8.0% for LeMI and ≤6.0% for LiMI at shelf life.

## Introduction

According to the International Council for Harmonization (ICH) Q3B(R2) guidelines, an impurity is defined as being any component of a drug product that is not the drug substance or an excipient in the drug product.^1^ Impurities in a drug product can arise due to different reasons, but regardless of their origin, even small amounts of impurities could influence the quality, safety, and/or efficacy of pharmaceutical products.^2^ Since complete elimination of impurities is often not possible, the concept of threshold of toxicological concern was developed.^3^

Several regulations related to impurities in drug applications have been introduced by the European Medicines Agency (EMA), the US Food and Drug Administration (FDA), and the ICH.^1^^,^^3^^,^^4^ Although authorities focus mainly on guidance for new drug substances and drug products, marketing authorization holders are required to also monitor degradation products and conduct stability studies in their authorized products, to identify any impurity/degradation product that exceeds pre-specified identification thresholds.

ADVANZ PHARMA has developed new stability-indicating related substances methods to quantify impurities in levothyroxine (LeMI) and liothyronine (LiMI) tablets, which were validated according to ICH Q2(R1) guidelines.^5^ Following the introduction of these new methods, an unknown impurity (with a relative retention time [RRT] of 0.92 and 0.90 in the case of LeMI tablets and LiMI tablets, respectively) was observed in finished product samples, which, from 6 months onwards, exceeded the corresponding qualification threshold (1.0%) specified by the ICH Q3B(R2) guideline.^1^ Liquid chromatography–mass spectrometry (LCMS) testing revealed that the impurities were formed through a Maillard condensation reaction between LeMI or LiMI and lactose.

The Maillard reaction, a form of nonenzymatic browning, is a chemical interaction that occurs generally between a reducing carbohydrate and the amino group of a peptide or protein, under certain reaction conditions.^6^^,^^7^ The main promoter of the Maillard reaction is heat, but increased humidity and alkaline pH can also intensify the reaction.^7^ In drug products formulated as tablets, a Maillard reaction can occur during storage between the amine groups of active pharmaceutical ingredients and the carbonyl groups contained by the open-chain aldehyde forms of reducing carbohydrates used as an excipient.^8–10^ As a consequence, the amount of the therapeutic agent may decrease, drug bioavailability may be altered, and the resultant compounds may potentially be toxic.^6^^,^^7^^,^^11^

Currently, there is no defined specification for Maillard condensation product/Maillard impurity levels for drugs in the European Pharmacopoeia or the British Pharmacopoeia. Following discussions with the Medicines and Healthcare products Regulatory Agency (MHRA), ADVANZ PHARMA has committed to perform a comprehensive nonclinical toxicological assessment including *in silico*, *in vitro*, and *in vivo* tests, in order to establish the toxicity profile of these impurities in humans and to define and propose appropriate shelf-life specification limits for LeMI and LiMI.

## Material and methods

### *In silico* toxicological assessment

**Fig. 1:**
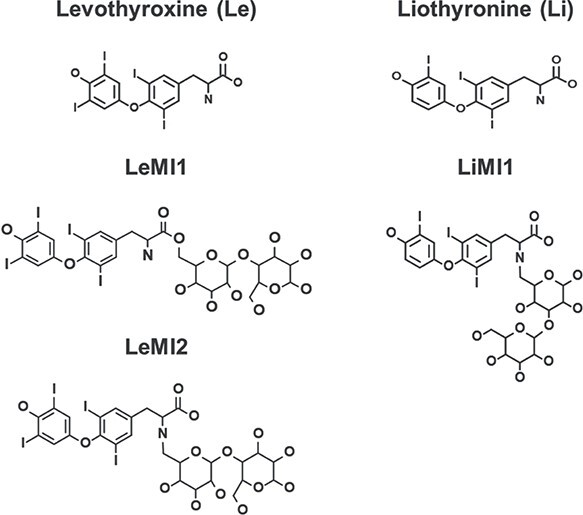
Two-dimensional representation of the chemical structures of levothyroxine, liothyronine, and of their potential Maillard condensation products, LeMI1, LeMI2, and LiMI1, used for *in silico* toxicological assessments. LeMI1, levothyroxine-lactose Maillard impurity, potential structure no. 1; LeMI2, levothyroxine-lactose Maillard impurity, potential structure no. 2; LiMI, liothyronine-lactose Maillard impurity, potential structure no. 1.

Two *in silico* methods (one statistical—Leadscope, and one rule-based—Derek Nexus) were used to explore the potential toxicity of both impurities and to provide an overall risk assessment by also considering the total daily exposure and the known toxicity profile of LeMI and LiMI. Chemical structures of the processed compounds are depicted in Fig. 1.

*Leadscope* is a quantitative structural activity relationship model that predicts toxicity by comparing the entered structure with empirical data and subsequently assigns a probability value based on likelihood of occurrence. Details of the Leadscope Model Applier Versions and suites used for these analyses are presented in Supplementary Table 1. Datasets were processed against the following endpoints: clastogenicity *in vitro*, clastogenicity *in vivo*, and gene mutation.

*Derek Nexus 5.0.2* is a rule-based expert system, which assigns various levels of non-numeric likelihood to its predictions ranging from “certain” to “contradicted,” via “probable,” “plausible,” “equivocal,” “doubted,” “improbable,” “impossible,” and “open.” Details for this analysis are included in Supplementary Table 2. Datasets were processed against the following endpoints: carcinogenicity, genotoxicity (including mutagenicity and chromosome damage), irritation, miscellaneous endpoints, neurotoxicity, organ toxicity, reproductive toxicity, respiratory sensitization, and skin sensitization. The presentation and analysis of these results focused initially on mutagenic and genotoxic endpoints, followed by other endpoints triggered. This order was chosen as the genetic toxicity endpoint exhibits the lowest threshold of effect.

### Synthesis of the impurities

Due to technical constraints represented by the large volume of tablets required to reach the concentrations of LeMI and LiMI planned to be used for *in vitro* and *in vivo* toxicological studies, the 2 impurities were synthetized by ADVANZ PHARMA approved service providers. All reagents were sourced from approved suppliers.

**Fig. 2:**
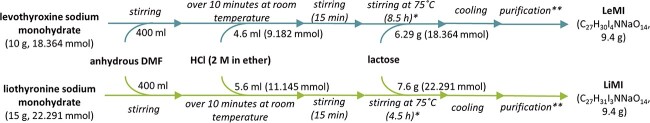
Synthetic procedures for LeMI and LiMI. ^*^To select the optimal duration of this step, the reaction was initially carried out over different time periods ranging from 1 to 10 h, and the duration that resulted in a maximum conversion to the targeted product was identified. ^*^^*^Details of the purification process are described in Supplementary File 1. DMF, dimethylformamide; LeMI, levothyroxine-lactose Maillard impurity; LiMI, liothyronine-lactose Maillard impurity.

**Fig. 3:**
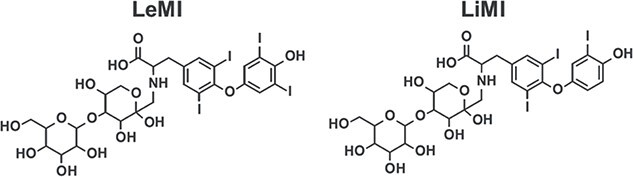
Chemical structures of the 2 synthetized Maillard condensation products (LeMI and LiMI, keto form), as determined by LCMS and NMR spectroscopy. LeMI, levothyroxine-lactose Maillard impurity; LiMI, liothyronine-lactose Maillard impurity; LCMS, liquid chromatography-mass spectrometry; NMR, nuclear magnetic resonance.

Synthesis of LeMI and LiMI was carried out separately, in an argon atmosphere. Figure 2 describes the details of the synthesis processes for both impurities. Chemical structures of the synthetized impurities are depicted in Fig. 3. The resulting products, in the form of pale yellow powders, were pure by nuclear magnetic resonance spectroscopy, with >93% and > 95% purity by high-performance liquid chromatography, for LeMI and LiMI, respectively. Expected structures of LeMI and LiMI were further confirmed by LCMS, as well as by ^1^H, ^13^C, and 2D nuclear magnetic resonance spectroscopy. The monoisotopic molecular weights determined by LCMS were 1100.8 for LeMI and 974.9 for LiMI, in line with each impurity’s nominal molecular weight (1101 for LeMI, 975 for LiMI).

### *In vitro* toxicological assessment

The mutagenic potential of LeMI and LiMI was evaluated through the Ames test, carried out both in the presence and absence of a metabolic activation system (Aroclor 1254-induced rat liver S9 homogenate). Dimethyl sulfoxide (DMSO) was used as a vehicle for both test items. Interpretation of the test results was performed as described in Supplementary Table 3.

*A preliminary cytotoxicity test* was carried out to establish the concentration range for the mutagenicity study (Supplementary Table 4). The test strain was represented by *Salmonella typhimurium* strain TA 100. For both impurities, 7 different concentrations were tested, with the concentration of 5000 μg/plate being the highest.

The *mutagenicity study* included 2 separate trials (Supplementary Table 4). Tester strains were represented by the *S. typhimurium* strains TA 98, TA 100, TA 1535, and TA 1537 and the *Escherichia coli* strain WP2 uvrA (pKM101), as recommended by the OECD Guideline No. 471^12^ and the ICH-harmonized guidances on genotoxicity testing of pharmaceuticals.^13^ Based on the results of the cytotoxicity test, 5000 μg/plate was selected as the highest concentration; 4 additional concentrations of the test items were selected to meet guideline requirements.^12^

### *In vivo* toxicological assessment

*In vivo* toxicological assessment of LeMI and LiMI was carried out by performing 7-day dose range finding (DRF) and 90-day dose repeat studies. The rat was chosen as the animal model as it is an accepted rodent species for preclinical toxicity testing by regulatory agencies. The current state of scientific knowledge and the applicable guidelines for 7-day DRF^14^^,^^15^ and 90-day repeat dose studies^1^^,^^16^ did not provide acceptable alternatives, *in vitro* or otherwise, to the use of live animals to accomplish the purpose of these studies.

All animals were handled humanely with due regard for their welfare. Every effort was made to minimize and to eliminate pain and suffering of animals in the study. Care of animals complied with the recommendations of Association for Assessment and Accreditation of Laboratory Animal Care International and Committee for the Purpose of Control and Supervision of Experiments on Animals, Government of India. Details regarding the housing and husbandry of the test animals are described in Supplementary File 2 (7-day DRF study) and Supplementary File 3 (90-day dose repeat study). The studies were designed to use the minimum number of animals possible. Approval for the experiments was obtained from the Institutional Animal Ethics Committee (IAEC Protocol No: SYNGENE/IAEC/992/09-2018).

Data collected from both studies were evaluated using the Levene’s Test for homogeneity of variances. Homogeneous data were compared using analysis of variance; in case of statistical significance, pairwise comparisons of each treated group with the control group were made using a Dunnett test. Statistical significance was set at a 5% level.

**Fig. 4:**
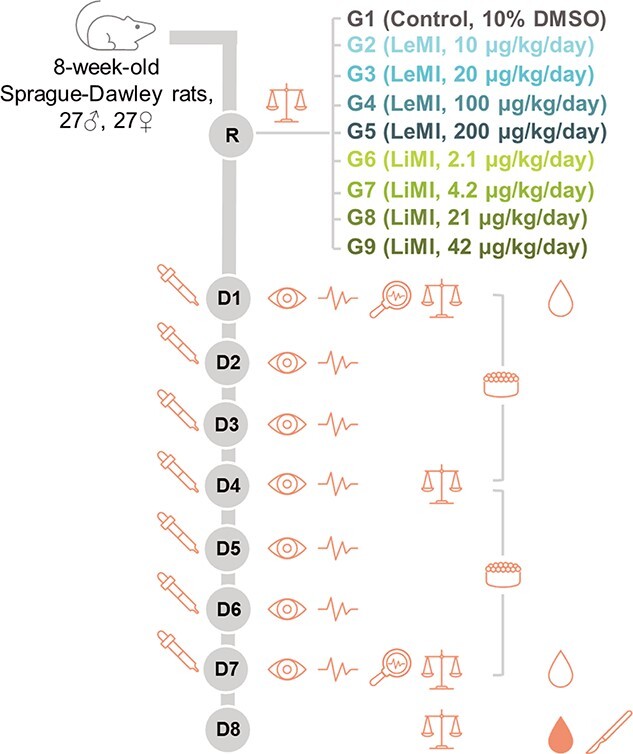
7-day dose range finding study design 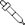, study treatment/control (5 mL/kg); 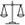, body weight measurement; 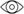, morbidity/mortality check, twice daily; 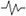, observation for clinical signs of toxicity, at least once a day; 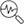, detailed clinical examination (e.g. changes in skin, fur, eyes and mucous membranes and in respiratory, circulatory, autonomic and central nervous system, somatomotor activity, and behavioral pattern); 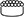, average individual food consumption (g/animal/day) = Food offered (g)–Food leftover (g)/(No. of rats in cage x No. of days); 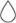, blood collection for bioanalysis and toxicokinetic analysis (LeMi and LiMI plasma levels, from 3 rats/sex/time point); 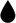, blood collection for hematology, coagulation, and clinical chemistry evaluations (details in Supplementary File 4); 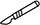, necropsy (details in Supplementary File 5); R, randomization using Pristima (based on body weight); G, group containing 3 rats/sex/group; DMSO, dimethyl sulfoxide; LeMI, levothyroxine-lactose Maillard impurity; LiMI, liothyronine-lactose Maillard impurity; D, day.

*The 7-day DRF study* evaluated the toxicity and toxicokinetic of 4 different dosages of LeMi (Groups [G] 2–5) and LiMI (G6–9) when administered once daily by oral gavage to rats for a period of 7 days. 10% DMSO was used as a vehicle to prepare the test item formulations and as control (G1). The study design is presented in Fig. 4.

For each test item, the 4 dose levels were selected in consultation with the sponsor and were based on the ICH Q3B(R2)^1^ qualification threshold; for drug products with daily doses of <10 mg, the qualification threshold is represented by 1.0% or 50 μg total daily intake, whichever is lower. Considering that the maximum daily doses for LeMI and LiMI are 200 and 60 μg, and the highest anticipated amount of LeMI/LiMI is 7%, the qualification threshold was calculated as 14 μg/day for LeMI and 4.2 μg/day for LiMI. The lowest doses tested were below the qualification thresholds for both impurities.

**Fig. 5:**
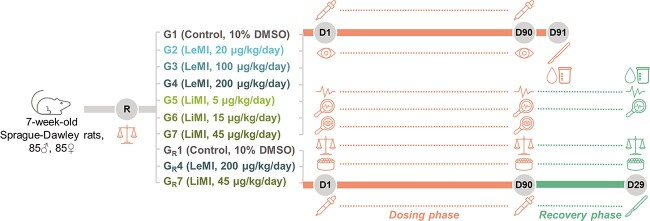
90-day dose repeat study design 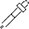, study treatment/control (5 mL/kg); 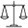, body weight measurement, once weekly; 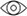, morbidity/mortality check, twice daily; 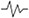, observation for clinical signs of toxicity, at least once a day; 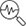, detailed clinical examination, once weekly (e.g. changes in skin, fur, eyes and mucous membranes and in respiratory, circulatory, autonomic and central nervous system, somatomotor activity, and behavioral pattern); 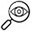, ophthalmic examination (only for groups G1, G4, and G7 at D90); 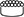, average food consumption (g/cage/day), weekly; 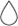, blood collection for hematology, coagulation and clinical chemistry evaluations (details in Supplementary File 4); 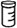, urine collection for urinalysis (details in Supplementary File 4); 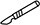, necropsy (details in Supplementary File 6); R, randomization using Pristima (based on body weight); G, main toxicity group containing 10 rats/sex/group; G_R_, recovery group, containing 5 rats/sex/group; DMSO, dimethyl sulfoxide; LeMI, levothyroxine-lactose Maillard impurity; LiMI, liothyronine-lactose Maillard impurity; D, day.

The *90-day dose repeat study* evaluated the toxicity of 3 different dosages of LeMI (G2–4) and LiMI (G5–7) administered once daily by oral gavage to rats for a period of 90 days and assessed the reversibility, persistence, or delayed occurrence of toxic effects after a 28-day recovery period in control and high dose groups (G_R_1, G_R_4, G_R_7). 10% DMSO was used as a vehicle to prepare the test item formulations and as control (G1 and G_R_1). The study design is presented in Fig. 5.

For each test item, the 3 dose levels were selected based on the results of the 7-day DRF study. The doses of LeMi and LiMI used in this study are equivalent to 4x (20 μg/kg), 20x (100 μg/kg), and 40x (200 μg/kg) and 1x (5 μg/kg), 3x (15 μg/kg), and 9x (45 μg/kg) human exposure based on body surface area, respectively. The high dose was expected to produce minimal toxicity. The other concentrations were selected to assess a dose response for any observed effects and to establish a no-observed-adverse-effect-level (NOAEL).

## Results

### *In silico* toxicological assessment

#### Leadscope analysis

Results of the Leadscope analysis are presented in Table 1.

**Table 1:** Results of the Leadscope analysis for levothyroxine, liothyronine, and their Maillard condensation products.

Structures tested	Prediction calls			Out of domain	Models with positive prediction calls
	Positive	Negative	Indeterminate		
Le	3	10	0	3	*In vitro* SCE (other cells) *In vivo* chromosome aberration (rat) *In vivo* micronucleus (rodent)
LeMI1	2	11	1	2	*In vitro* SCE (other cells) *In vivo* micronucleus (rodent)
LeMI2	0	11	1	4	
Li	2	9	0	5	*In vitro* SCE (other cells) *In vivo* chromosome aberration (rat)
LiMI1	0	12	0	4	

Le, levothyroxine; LeMI1, levothyroxine-lactose Maillard impurity, potential structure no. 1; LeMI2, levothyroxine-lactose Maillard impurity, potential structure no. 2; Li, liothyronine; LiMI1, liothyronine-lactose Maillard impurity, potential structure no. 1; SCE, sister chromatid exchange

Examination of the training sets of the major positive contributing features suggests that these prediction calls are false positive results and of little relevance to these structures. This position is further confirmed by the evidence for a lack of genetic toxicity of LeMI and LiMI at clinically relevant doses.

Overall, these results suggest that neither LeMI and its 2 potential Maillard condensation products nor LiMI and its potential Maillard condensation product should be considered bacterial mutagens or *in vitro* or *in vivo* clastogens.

#### Derek Nexus analysis

LeMI and its 2 potential Maillard condensation products as well as LiMI and its potential Maillard condensation product triggered no human alerts for carcinogenicity, chromosome damage, genotoxicity, or mutagenicity. The Derek Nexus software indicated that none of these structures contained any misclassified or unclassified features and as such were deemed inactive for *in vitro* bacterial mutagenicity.

A “plausible” alert in humans for thyroid toxicity was triggered by all 5 structures, due to an aromatic iodo moiety. In other nonhuman mammals, this alert was extrapolated to carcinogenicity, as thyroid toxicity is associated with the formation of tumors of the thyroid and pituitary glands. However, humans are much less susceptible to this effect than rodents.^17–19^ This alert is clearly a false positive in the case of LeMI and LiMI, as these are synthetic analogs of an endogenous product; therefore, the occurrence of this alert was not considered relevant. As a result, this extrapolated alert for carcinogenicity was given an “equivocal” likelihood in humans for all structures analyzed.

Overall, evidence from empirical and *in silico* sources, coupled with exposure considerations, suggests that the presence of these impurities at low oral exposure levels (≤30 μg/day/patient [up to 10%] for LeMI and ≤ 4.2 μg/day/patient [up to 7%] for LiMI) should not be associated with additional risk to the patient (over and above that of LeMI or LiMI).

### *In vitro* toxicological assessment (Ames test)

In the preliminary cytotoxicity study, the bacterial background lawn of tester strain TA 100 and the number of revertant colonies in all the tested concentrations were comparable with vehicle control group for both LeMI and LiMI (Supplementary Table 5).

In Trial 1 of the mutagenicity study, the growth of the bacterial background lawn (data not shown) and mean number of revertant colonies were comparable with the vehicle control plates, in the presence or absence of metabolic activation, for both LeMI (Supplementary Table 6) and LiMI (Supplementary Table 7), for all tester strains. Results of Trial 2 were similar to those observed in mutagenicity study Trial 1 (Supplementary Tables 6 and 7).

Based on these results, it was concluded that neither LeMI nor LiMI was cytotoxic or mutagenic at up to 5000 μg/plate, in the presence and absence of metabolic activation.

### *In vivo* toxicological assessment

#### 7-day DRF study

No morbidity or mortality was observed in any of the 9 study groups. There were no test item-related systemic clinical signs of toxicity in any of the rats that were administered LeMI and LiMI, regardless of the dose received. No test items-related changes were observed in hematology, coagulation, and clinical chemistry parameters. The few minor differences that were observed at multiple dose levels were considered incidental due to lack of a dose–response relationship (data not shown). There were no test item-related changes in body weight, food consumption, and organ weights (data not shown). A lower weight of the thyroid and parathyroid glands compared with the control group was noted in around one-third of the treated animals (data not shown); this change exhibited no dose–response relationship and was considered to be related to the pharmacological activity of LeMI and LiMI.

There were no test item-related gross changes. A minimal or mild increase in colloid in the follicles of the thyroid gland of males and females was noted. For LeMI, this finding was observed only in females at dose levels of ≥100 μg/kg/day, while for LiMI, it was observed in males at dose levels of ≥21 μg/kg/day and in females at dose levels of ≥4.2 μg/kg/day. This change was considered secondary to the pharmacologic activity of the test items.

LeMi and LiMI plasma concentrations were below the quantification limit at all time points (data not shown); therefore, toxicokinetic evaluation was not performed.

#### 90-day dose repeat study

During both the dosing and the recovery phases, there were no treatment-related morbidity or mortality, clinical signs of systemic toxicity, or ophthalmoscopic findings in any of the 10 study groups. No test item-related changes in body weight, body weight gain, and food consumption were recorded. There were no test item-related gross changes, changes in hematologic and coagulation parameters, or changes in urinalysis parameters in animals administered LeMI or LiMI, regardless of the dose received.

Minor changes in clinical chemistry parameters were observed in certain groups at the terminal or recovery sacrifices (data not shown); these changes were not considered toxicologically significant, as they were of minimal magnitude, within historical control range, occurred only in one sex, and were inconsistent between scheduled sacrifices.

Minimal increase in thyroid weights and microscopic change of minimal or mild increase in colloid of thyroid follicles were observed at one or more dose levels at the terminal sacrifice (data not shown). These changes were considered likely to be related to the pharmacological action of the test items; all changes reverted completely at the end of the 28-day recovery period. All other changes in organ weight parameters and microscopic changes were considered incidental (data not shown).

The NOAEL determined in Sprague–Dawley rats was 200 μg/kg/day for LeMI and 45 μg/kg/day for LiMI.

### Proposal of new safe specifications limits

Based on the results reported above, safe limits for LeMI and LiMI were proposed, to support changes to the specification for related substances at shelf life (Table 2).

**Table 2:** Proposal for safe limits of LeMI and LiMI in levothyroxine and liothyronine tablets.

	Release specification	Shelf-life specification
LeMI (RRT .92)	NMT 3.0%	NMT 8.0%
LiMI (RRT .90)	NMT 1.0%	NMT 6.0%

LeMI, levothyroxine-lactose Maillard impurity; LiMI, liothyronine-lactose Maillard impurity; RRT, relative retention time; NMT, not more than

Taking into account a maximum recommended human daily dose of 200 μg/day for LeMI and of 60 μg/day for LiMI, a limit of not more than (NMT) 8.0% for LeMI and 6.0% for LiMI will correspond to daily exposures of 16 and 3.6 μg/day, respectively. The NOAEL doses of 200 μg/kg/day for LeMI and 45 μg/kg/day for LiMI established in the 90-day dose repeat study in rats represent approximately 120–122 times the maximum daily exposures of LeMI and LiMI, based on body surface area (μg/m^2^).

## Discussion

The ICH M7 guideline on assessment and control of DNA reactive (mutagenic) impurities in pharmaceuticals to limit potential carcinogenic risk^3^ allows for the replacement of a bacterial reverse mutation assay by use of 2 complementary *in silico* methodologies (one expert rule-based and one statistical-based). The choice of *in silico* methods used in the toxicological assessment of both impurities is aligned with the recommendations of the ICH M7 guideline. The combined use of Derek Nexus and Leadscope has been reported to result in significantly improved accuracy, sensitivity, and specificity compared with use of one program alone.^20^ Overall, the evidence from empirical and *in silico* sources indicates that the impurities should not be considered carcinogenic, genotoxic, or mutagenic *in vitro* and *in vivo*, and so these impurities should be categorized as class 5 under ICH M7.

Subsequently, impurities were synthetized for *in vitro* and *in vivo* toxicological analyses. Both impurities were assessed to possess noncarcinogenic, non-genotoxic, non-mutagenic, or non-cytotoxic potential *in vitro* and *in vivo*. Furthermore, *in vivo* studies revealed safety margins of >120 times the maximum human daily exposure to LeMi or LiMI. Based on these results, we have proposed to increase the limits of Maillard condensation products to NMT 8.0% for LeMI and 6.0% for LiMI at shelf life. At the time of drafting this manuscript, the proposal for LeMI was already approved by the MHRA, while the application for LiMI was still under assessment.

Although excipients should remain inert throughout the shelf life of a drug product, depending on their chemical structure and on that of the active ingredient, interactions between them are possible. The Maillard reaction, which occurs between a primary amine and lactose,^6^ is an example of such an interaction. The reactivity of the Maillard reaction may vary depending on the concentration of active substance, the concentration and characteristics of the lactose used, and also with other factors that intervene during processing and storage, such as heat, pressure, or water content of the final product.^6^^,^^21^^,^^22^

Currently, there is no specification for Maillard condensation product impurities in the European or the British Pharmacopoeia. Based on the toxicological data reported here and the wide safety margin of LeMI and LiMI observed in the animal studies, a new limitation for Maillard condensation impurities can be established. Currently, ADVANZ PHARMA is jointly working with the British Pharmacopoeia for implementing these new limits. Defined limits for Maillard condensation product impurity will be very useful as this type of impurity can occur in other lactose containing products as well.^8^^,^^9^

Due to technical constrains related to the large quantity of tablets needed to obtain the pre-specified concentrations of LeMI and LiMI for *in vitro* and *in vivo* tests, the impurities were synthetized, which can be regarded as a potential limitation of the study. However, structural analysis confirmed that the synthetized impurities were structurally identical to those identified in LeMI and LiMI tablets. The potential LeMI and LiMI structures used for *in silico* analyses differed slightly from those of the synthetized compounds. However, *in silico* results can still be considered as relevant for the synthetized impurities, as confirmed by the results of the *in vitro* and *in vivo* studies.

## Conclusions

No concerns regarding the toxicity, carcinogenicity, or mutagenicity of LeMI and LiMI have been raised in either of the nonclinical tests performed. Based on the toxicological data presented here and the wide safety margin of the Maillard condensation impurities achieved in animal studies with regards to human daily exposure, the human safety of LeMI and LiMI was confirmed, and a new limitation for these impurities can be established.

## Supplementary Material

MaillardImpurities_SupplementaryMaterial_tfac047
