# Supplementary material for: Preclinical toxicological assessment of levothyroxine and liothyronine Maillard impurities
Source: Toxicol Res (Camb). 2022 Aug 13;11(5):743–9. doi: 10.1093/toxres/tfac047 (PMC9618099; doi:10.1093/toxres/tfac047)
Supplement: MaillardImpurities_SupplementaryMaterial_tfac047 [file maillardimpurities_supplementarymaterial_tfac047.docx]

**Supplementary files**

**Supplementary Table 1. Leadscope Model Applier Versions and suites used for prediction**

| **Structures analyzed** | **Model Applier Version** | **Interpretation of prediction probabilities** | | |
| --- | --- | --- | --- | --- |
|  |  | **Positive** | **Negative** | **Indeterminate** |
| ***Le, LeMI1 and LeMI2*** | 2.0.3, Genetic Toxicity Suite* | ≥0.6 | <0.4 | 0.4–<0.6 |
| ***Li and LiMI1*** | 2.2.2, Genetox Statistical Model Suite* |  |  |  |
|  | - *Salmonella* Mut v3 and *E. coli*–*Sal* 102 A-T Mut v1 models | ≥0.6 | <0.4 | 0.4–<0.6 |
|  | - Other models | ≥0.5 | <0.5 | NA |
| Le, levothyroxine; LeMI1, levothyroxine-lactose Maillard impurity, potential structure no. 1; LeMI2, levothyroxine-lactose Maillard impurity, potential structure no. 2; Li, liothyronine; LiMI1, liothyronine-lactose Maillard impurity, potential structure no. 1; AT, adenine-thymine base pair; NA, not applicable  *developed through a Research Collaboration Agreement with the United States Food and Drug Administration | | | | |

**Supplementary Table 2. Details of the Derek Nexus analysis**

| **Structures analyzed** | **Knowledge base** | **Interpretation of predictions** | **Species of interest** | **Processing constraints** |
| --- | --- | --- | --- | --- |
| ***Le, LeMI1 and LeMI2*** | 2014 1.0 | “Plausible”: the structure has activated an alert for the endpoint in question and there is sufficient information in the database to support the prediction  “Equivocal”: there is an equal weight of evidence for and against the proposition  “Open”: there is no evidence that supports or opposes the proposition | *E. coli*, *S. typhimurium*, bacterium, dog, guinea pig, hamster, human, mammal, monkey, mouse, primate, rabbit, rat and rodent | The option to perceive tautomers was selected. |
| ***Li and LiMI1*** | 2015 2.0 |  |  |  |
| Le, levothyroxine; LeMI1, levothyroxine-lactose Maillard impurity, potential structure no. 1; LeMI2, levothyroxine-lactose Maillard impurity, potential structure no. 2; Li, liothyronine; LiMI1, liothyronine-lactose Maillard impurity, potential structure no. 1 | | | | |

**Supplementary Table 3. Interpretation of the Ames test results used for the *in vitro* study**

| **Interpretation** | **Result** |
| --- | --- |
| **Mutagenic** | - a concentration-related increase of the revertant count/plate in ≥1 test strain over ≥2 increasing concentrations of the test item - the result is considered positive if the mean number of revertants at the peak of the concentration is: - ≥2 × the mean number of negative control revertants, for strains TA 98, TA 100 and WP2 uvrA (pKM101) - ≥3 × the mean number of negative control revertants, for strains TA 1535 and TA 1537 |
| **Equivocal** | - a biologically relevant increase in the revertant count that partially meets the criteria for the evaluation as positive (a concentration-related increase that does not achieve the thresholds cited above or a non-concentration related increase that is equal to or greater than the thresholds cited above) |
| **Non-mutagenic** | - neither mutagenic nor equivocal |

**Supplementary Table 4. Ames test methodology used to evaluate the *in vitro* mutagenic potential of LeMI and LiMI**

| **Substance(s) tested** | **Test item concentrations (μg/plate)** | **Control(s)** | **No. of replicates** | **Culture media** | **Incubation** |
| --- | --- | --- | --- | --- | --- |
| ***Cytotoxicity test*** | | | | | |
| **LeMI**  **and**  **LiMI** | 78.125  156.25  312.5  625  1250  2500  5000 | vehicle control (DMSO) | duplicate | Step 1: Oxoid nutrient broth no. 2 (overnight culture) → 100 μL added to Step 2  Step 2: 500 μL S9 mix*/PBS, 100 μL test item solution/control and 2 mL amino acid-containing soft agar (histidine and biotin for *S. typhimurium*, tryptophan for *E. coli*) → poured onto VB agar plates | Step 1: 37±1 ˚C  18 h  Step 2: 37±1 ˚C  68 h |
| ***Mutagenicity test – Trial 1 (plate incorporation method)*** | | | | | |
| **LeMI** | 50  159  501  1582  5000 | vehicle control (DMSO)  positive control (strain specific) | triplicate | Step 1: Oxoid nutrient broth no. 2 (overnight culture) → 100 μL added to Step 2  Step 2: 500 μL S9 mix*/PBS, 100 μL test item solution/control and 2 mL amino acid-containing soft agar (histidine and biotin for *S. typhimurium*, tryptophan for *E. coli*) → poured onto VB agar plates | Step 1: 37±1 ˚C  18 h  Step 2: 37±1 ˚C  65 h |
| **LiMI** | 99  265  699  1869  5000 |  |  |  | Step 1: 37±1 ˚C  18 h  Step 2: 37±1 ˚C  50 h |
| ***Mutagenicity test – Trial 2 (pre-incubation method: 20 minutes at 37±1 ˚C under shaking)*** | | | | | |
| **LeMI** | 50  159  501  1582  5000 | vehicle control (DMSO)  positive control (strain specific) | triplicate | Step 1: Oxoid nutrient broth no. 2 (overnight culture) → 100 μL added to Step 2  Step 2: 500 μL S9 mix*/PBS, 100 μL test item solution/control and 2 mL amino acid-containing soft agar (histidine and biotin for *S. typhimurium*, tryptophan for *E. coli*) → poured onto VB agar plates | Step 1: 37±1 ˚C  17 h  Step 2: 37±1 ˚C  ~67 h |
| **LiMI** | 99  265  699  1869  5000 |  |  |  |  |
| LeMI, levothyroxine-lactose Maillard impurity; LiMI, liothyronine-lactose Maillard impurity; DMSO, dimethyl sulfoxide; PBS, phosphate buffered saline; VB, Vogel-Bonner  *Prepared using PBS, nicotinamide adenine dinucleotide phosphate, glucose-6-phosphate, solution of salts (MgCl_2_, KCl). The concentration of the S9 mix was 10% v/v for the preliminary cytotoxicity test and mutagenicity study Trial 1 and 5% v/v for the mutagenicity study Trial 2. | | | | | |

**Supplementary Table 5. Results of the preliminary cytotoxicity test for the *S. typhimurium* TA 100 tester strain treated with different concentrations of LeMI and LiMI, in the presence and absence of metabolic activation**

| **Concentration (μg/plate)** | **Number of revertants/plate*,**  **mean±SD (FI)** | | **Bacterial background lawn** | | **Precipitation**** | |
| --- | --- | --- | --- | --- | --- | --- |
|  | *+ MA* | *– MA* | *+ MA* | *– MA* | *+ MA* | *– MA* |
| **LeMI** | | | | | | |
| **Vehicle control (DMSO)** | 131±4 **(NA)** | 131±4 **(NA)** | 4+ | 4+ | No | No |
| **78.125** | 130±1 **(1)** | 130±1 **(1)** | 4+ | 4+ | No | No |
| **156.25** | 129±3 **(1)** | 131±2 **(1)** | 4+ | 4+ | No | No |
| **312.5** | 131±1 **(1)** | 128±1 **(1)** | 4+ | 4+ | No | No |
| **625** | 128±2 **(1)** | 130±1 **(1)** | 4+ | 4+ | No | No |
| **1250** | 130±1 **(1)** | 130±1 **(1)** | 4+ | 4+ | No | No |
| **2500** | 131±1 **(1)** | 131±2 **(1)** | 4+ | 4+ | Yes | Yes |
| **5000** | 130±4 **(1)** | 130±1 **(1)** | 4+ | 4+ | Yes | Yes |
| **LiMI** | | | | | | |
| **Vehicle control (DMSO)** | 126±1 **(NA)** | 125±1 **(NA)** | 4+ | 4+ | No | No |
| **78.125** | 124±1 **(1)** | 124±1 **(1)** | 4+ | 4+ | No | No |
| **156.25** | 124±4 **(1)** | 124±2 **(1)** | 4+ | 4+ | No | No |
| **312.5** | 125±1 **(1)** | 125±2 **(1)** | 4+ | 4+ | No | No |
| **625** | 125±2 **(1)** | 124±1 **(1)** | 4+ | 4+ | No | No |
| **1250** | 123±3 **(1)** | 124±1 **(1)** | 4+ | 4+ | No | No |
| **2500** | 123±4 **(1)** | 123±2 **(1)** | 4+ | 4+ | Yes | Yes |
| **5000** | 125±4 **(1)** | 125±2 **(1)** | 4+ | 4+ | Yes | Yes |
| LeMI, levothyroxine-lactose Maillard impurity; LiMI, liothyronine-lactose Maillard impurity; DMSO, dimethyl sulfoxide; SD, standard deviation; NA, not applicable; FI, fold increase; MA, metabolic activation; 4+, thick lawn  *The values were rounded to a whole number.  **Precipitation which did not interfere with the counting of colonies  FI = Mean of individual revertant colonies in the treated group / Mean of individual revertant colonies in the vehicle control group | | | | | | |

**Supplementary Table 6. Number of revertant colonies for the tester strains used in the mutagenicity study, treated with different concentrations of LeMI, in the presence and absence of metabolic activation**

| **Concentration (μg/plate)** | | **Number of revertants/plate*** | | | | | | | | | |
| --- | --- | --- | --- | --- | --- | --- | --- | --- | --- | --- | --- |
|  |  | **TA 98** | | **TA 100** | | **TA 1535** | | **TA 1537** | | **WP2 uvrA**  **(pKM101)** | |
|  |  | ***Mean***  **±*SD*** | ***FI*** | ***Mean* ±*SD*** | ***FI*** | ***Mean* ±*SD*** | ***FI*** | ***Mean* ±*SD*** | ***FI*** | ***Mean* ±*SD*** | ***FI*** |
| ***Mutagenicity test Trial 1*** | | | | | | | | | | | |
| **Vehicle control (DMSO)** | *+ MA* | 27±3 | **NA** | 120±2 | **NA** | 11±2 | **NA** | 7±2 | **NA** | 122±3 | **NA** |
|  | *– MA* | 29±1 | **NA** | 122±2 | **NA** | 13±2 | **NA** | 7±1 | **NA** | 124±5 | **NA** |
| **50** | *+ MA* | 29±4 | **1** | 120±2 | **1** | 12±3 | **1** | 8±1 | **1** | 123±4 | **1** |
|  | *– MA* | 29±2 | **1** | 121±5 | **1** | 13±2 | **1** | 7±2 | **1** | 125±4 | **1** |
| **159** | *+ MA* | 30±2 | **1** | 120±2 | **1** | 12±3 | **1** | 7±3 | **1** | 123±2 | **1** |
|  | *– MA* | 27±3 | **1** | 121±3 | **1** | 13±1 | **1** | 8±1 | **1** | 125±2 | **1** |
| **501** | *+ MA* | 28±2 | **1** | 121±4 | **1** | 11±2 | **1** | 7±2 | **1** | 122±4 | **1** |
|  | *– MA* | 28±2 | **1** | 122±1 | **1** | 13±3 | **1** | 7±1 | **1** | 126±1 | **1** |
| **1582** | *+ MA* | 27±3 | **1** | 120±2 | **1** | 12±2 | **1** | 8±3 | **1** | 122±3 | **1** |
|  | *– MA* | 28±2 | **1** | 121±4 | **1** | 12±2 | **1** | 7±2 | **1** | 125±2 | **1** |
| **5000** | *+ MA* | 29±3 | **1** | 120±2 | **1** | 11±1 | **1** | 7±2 | **1** | 122±2 | **1** |
|  | *– MA* | 29±1 | **1** | 122±1 | **1** | 13±1 | **1** | 7±1 | **1** | 124±5 | **1** |
| **Positive control** | *+ MA* | 652±14 | **23** | 1007±5 | **8** | 171±2 | **15** | 162±6 | **23** | 785±16 | **6** |
|  | *– MA* | 310±5 | **11** | 811±9 | **7** | 180±7 | **14** | 131±4 | **13** | 876±11 | **8** |
| ***Mutagenicity test Trial 2*** | | | | | | | | | | | |
| **Vehicle control (DMSO)** | *+ MA* | 28±3 | **NA** | 120±2 | **NA** | 10±2 | **NA** | 7±2 | **NA** | 123±2 | **NA** |
|  | *– MA* | 27±2 | **NA** | 120±3 | **NA** | 9±1 | **NA** | 7±2 | **NA** | 123±3 | **NA** |
| **99** | *+ MA* | 28±2 | **1** | 119±4 | **1** | 10±3 | **1** | 7±2 | **1** | 122±4 | **1** |
|  | *– MA* | 26±3 | **1** | 119±3 | **1** | 9±2 | **1** | 8±3 | **1** | 123±2 | **1** |
| **265** | *+ MA* | 28±2 | **1** | 120±2 | **1** | 11±1 | **1** | 7±2 | **1** | 122±3 | **1** |
|  | *– MA* | 27±1 | **1** | 120±2 | **1** | 9±1 | **1** | 8±1 | **1** | 122±4 | **1** |
| **699** | *+ MA* | 28±3 | **1** | 120±3 | **1** | 10±4 | **1** | 6±2 | **1** | 122±2 | **1** |
|  | *– MA* | 28±3 | **1** | 119±2 | **1** | 10±1 | **1** | 7±2 | **1** | 123±3 | **1** |
| **1869** | *+ MA* | 27±2 | **1** | 120±2 | **1** | 10±2 | **1** | 7±2 | **1** | 121±4 | **1** |
|  | *– MA* | 27±2 | **1** | 120±2 | **1** | 9±3 | **1** | 8±2 | **1** | 123±3 | **1** |
| **5000** | *+ MA* | 27±2 | **1** | 120±3 | **1** | 10±2 | **1** | 7±1 | **1** | 123±2 | **1** |
|  | *– MA* | 27±0 | **1** | 119±2 | **1** | 9±2 | **1** | 7±1 | **1** | 123±2 | **1** |
| **Positive control** | *+ MA* | 693±9 | **25** | 1000±4 | **8** | 177±4 | **17** | 138±5 | **19** | 804±7 | **7** |
|  | *– MA* | 352±9 | **13** | 900±7 | **8** | 167±6 | **19** | 157±6 | **21** | 874±7 | **7** |
| LeMI, levothyroxine-lactose Maillard impurity; SD, standard deviation; NA, not applicable; FI, fold increase; MA, metabolic activation  *The values were rounded off to whole number.  FI = Mean of individual revertant colonies in the treated group / Mean of individual revertant colonies in the vehicle control group | | | | | | | | | | | |

**Supplementary Table 7. Number of revertant colonies for the tester strains used in the mutagenicity study, treated with different concentrations of LiMI, in the presence and absence of metabolic activation**

| **Concentration (μg/plate)** | | **Number of revertants/plate*** | | | | | | | | | |
| --- | --- | --- | --- | --- | --- | --- | --- | --- | --- | --- | --- |
|  |  | **TA 98** | | **TA 100** | | **TA 1535** | | **TA 1537** | | **WP2 uvrA**  **(pKM101)** | |
|  |  | ***Mean***  **±*SD*** | ***FI*** | ***Mean* ±*SD*** | ***FI*** | ***Mean* ±*SD*** | ***FI*** | ***Mean* ±*SD*** | ***FI*** | ***Mean* ±*SD*** | ***FI*** |
| ***Mutagenicity test Trial 1*** | | | | | | | | | | | |
| **Vehicle control (DMSO)** | *+ MA* | 28±2 | **NA** | 119±3 | **NA** | 12±1 | **NA** | 9±1 | **NA** | 121±2 | **NA** |
|  | *– MA* | 29±1 | **NA** | 122±4 | **NA** | 12±2 | **NA** | 7±2 | **NA** | 123±4 | **NA** |
| **50** | *+ MA* | 26±2 | **1** | 118±3 | **1** | 12±2 | **1** | 9±1 | **1** | 120±3 | **1** |
|  | *– MA* | 28±3 | **1** | 122±3 | **1** | 12±1 | **1** | 7±3 | **1** | 123±5 | **1** |
| **159** | *+ MA* | 26±5 | **1** | 118±4 | **1** | 12±2 | **1** | 9±1 | **1** | 119±1 | **1** |
|  | *– MA* | 28±2 | **1** | 123±3 | **1** | 12±2 | **1** | 8±2 | **1** | 123±3 | **1** |
| **501** | *+ MA* | 27±3 | **1** | 119±3 | **1** | 12±2 | **1** | 8±1 | **1** | 120±2 | **1** |
|  | *– MA* | 28±2 | **1** | 122±2 | **1** | 12±3 | **1** | 7±3 | **1** | 123±2 | **1** |
| **1582** | *+ MA* | 28±2 | **1** | 118±2 | **1** | 12±2 | **1** | 9±1 | **1** | 120±2 | **1** |
|  | *– MA* | 28±1 | **1** | 122±4 | **1** | 12±1 | **1** | 7±2 | **1** | 123±4 | **1** |
| **5000** | *+ MA* | 27±3 | **1** | 118±3 | **1** | 12±2 | **1** | 9±1 | **1** | 120±2 | **1** |
|  | *– MA* | 29±1 | **1** | 122±4 | **1** | 12±3 | **1** | 7±1 | **1** | 123±3 | **1** |
| **Positive control** | *+ MA* | 675±7 | **23** | 1013±5 | **9** | 188±4 | **16** | 153±4 | **17** | 892±5 | **7** |
|  | *– MA* | 308±8 | **11** | 885±15 | **7** | 171±3 | **15** | 158±4 | **13** | 913±6 | **8** |
| ***Mutagenicity test Trial 2*** | | | | | | | | | | | |
| **Vehicle control (DMSO)** | *+ MA* | 28±2 | **NA** | 121±3 | **NA** | 9±1 | **NA** | 9±2 | **NA** | 119±1 | **NA** |
|  | *– MA* | 23±3 | **NA** | 120±3 | **NA** | 12±1 | **NA** | 9±2 | **NA** | 120±2 | **NA** |
| **99** | *+ MA* | 28±4 | **1** | 121±3 | **1** | 8±2 | **1** | 9±2 | **1** | 118±2 | **1** |
|  | *– MA* | 23±3 | **1** | 123±3 | **1** | 11±3 | **1** | 8±2 | **1** | 119±1 | **1** |
| **265** | *+ MA* | 28±2 | **1** | 122±1 | **1** | 8±3 | **1** | 10±1 | **1** | 119±2 | **1** |
|  | *– MA* | 24±1 | **1** | 123±3 | **1** | 11±2 | **1** | 8±2 | **1** | 119±3 | **1** |
| **699** | *+ MA* | 27±2 | **1** | 121±3 | **1** | 7±3 | **1** | 8±1 | **1** | 118±2 | **1** |
|  | *– MA* | 23±2 | **1** | 120±3 | **1** | 12±1 | **1** | 9±1 | **1** | 121±2 | **1** |
| **1869** | *+ MA* | 28±3 | **1** | 121±3 | **1** | 8±1 | **1** | 9±1 | **1** | 118±4 | **1** |
|  | *– MA* | 23±3 | **1** | 121±1 | **1** | 11±2 | **1** | 8±1 | **1** | 121±4 | **1** |
| **5000** | *+ MA* | 28±1 | **1** | 121±2 | **1** | 9±2 | **1** | 9±1 | **1** | 119±2 | **1** |
|  | *– MA* | 23±1 | **1** | 122±4 | **1** | 12±2 | **1** | 9±1 | **1** | 121±2 | **1** |
| **Positive control** | *+ MA* | 676±7 | **24** | 1008±6 | **8** | 178±5 | **21** | 149±3 | **17** | 807±8 | **7** |
|  | *– MA* | 303±9 | **13** | 890±22 | **7** | 178±4 | **15** | 164±5 | **18** | 911±9 | **8** |
| LiMI, liothyronine-lactose Maillard impurity; SD, standard deviation; NA, not applicable; FI, fold increase; MA, metabolic activation  *The values were rounded off to whole number.  FI = Mean of individual revertant colonies in the treated group / Mean of individual revertant colonies in the vehicle control group | | | | | | | | | | | |

**Supplementary File 1. Purification steps undertaken during the synthetic procedures of LeMI and LiMI**

1. After cooling, the resulting solution was mixed with ethyl acetate while stirring and was kept in the refrigerator overnight. Afterwards, the supernatant was decanted, and ethyl acetate was again added. Following stirring for 15 min, the supernatant was removed.
2. The resulting crude solid was further purified by adding an 85/10/5 solution of acetonitrile/water-0.1% trifluoroacetic acid (TFA) /anhydrous methanol for LeMI or acetonitrile/water-0.1% TFA (90/10) for LiMI, stirring for 1 h and centrifugation. The supernatant was decanted, and the washing process was repeated. The resulting solid, isolated by centrifugation, had a purity of ~90% (LeMI) or ~85% (LiMI).
3. Further purification was performed by reverse phase column chromatography (stationary phases: C18 silica, 90Å pore size, 80 g column; mobile phases: water [0.1% TFA], acetonitrile [0.1% TFA], 65% to 35% over 5 column volumes). Pure fractions were analysed by analytical HPLC and collected. The solvent was removed using rotary evaporation (no heat) followed by freeze drying.
4. The levothyroxine-lactose TFA and liothyronine-lactose TFA salts isolated as above underwent ion exchange followed by neutralization to obtain the title products in the form of sodium salts. For this, the TFA salts were mixed with 1 M HCl (2 equivalents), with the addition of dimethylformamide (0.6 ml/g of crude compound, to dissolve the salt) and stirred at room temperature for 1 h. The solvent and water were removed by rotary evaporation. The residue was cooled in an ice bath, treated with 1 M NaOH solution (3 equivalents) and allowed to come to room temperature while stirring for 1 h, after which the supernatant was decanted. Acetic acid (10% v/v water, 3 equivalents) was added and stirred for 1 h, then decanted; Na_2_CO_3_ solution (10%, v/v water, 3 equivalents) was subsequently added and stirred for 1 h. The mixture was then centrifuged, and the supernatant was decanted; washing was performed using 20% acetonitrile in water, followed by another centrifugation step. Acetonitrile was removed by rotary evaporation.

**Supplementary File 2. Housing and husbandry conditions for animals used in the 7-day dose range finding study**

The animals received for the study were acclimatized in the experimental room for at least 3 days prior to treatment initiation. The body weight variation of the animals did not exceed 20% of the mean body weight for each sex at the time of randomisation. Animals were maintained in a controlled environment with a temperature of 21.1–23.6 °C, a relative humidity of 40–49%, a light/dark cycle of 12 h each and 18 fresh air changes/h. Rats were housed as 2 animals of the same sex/cage in clean, sterilized polycarbonate rat cages covered with stainless steel grill tops. Animals were fed *ad libitum* (except during fasting, approximately 12–16 h prior to terminal blood collection) with rodent pellet diet. Potable autoclaved water filtered through reverse osmosis was also provided *ad libitum* to all animals. No known contaminants were present in the diet, water or bedding at levels that might interfere with this study.

**Supplementary File 3. Housing and husbandry conditions for animals used in the 90-day dose repeat study**

The animals received for the study were acclimatized in the experimental room for 5 (males) to 6 days (females) prior to treatment initiation. Animals were maintained in a controlled environment with a temperature of 19.1–24.6 °C, a relative humidity of 41–65%, a light/dark cycle of 12 h each and 21 fresh air changes/h. Rats were housed as 1 or 2 animals of the same sex per cage in clean, sterilized polycarbonate rat cages covered with stainless steel grill tops. Animals were fed *ad libitum* (except during overnight fasting prior to clinical pathology blood collection, ~12–16 h) with rodent pellet diet. Potable autoclaved water filtered through reverse osmosis was also provided *ad libitum* to all animals. No known contaminants were present in the diet, water or bedding at levels that might interfere with the study.

**Supplementary File 4. Paraclinical investigations in the 7-day dose range finding and 90-day dose repeat studies**

***For the 7-day dose range finding and the 90-day dose repeat studies:***

- Haematology: red blood cells, haemoglobin, haematocrit, mean corpuscular volume, mean corpuscular haemoglobin, mean corpuscular haemoglobin concentration, platelets, reticulocytes (absolute), white blood cells, neutrophils (absolute), lymphocytes (absolute), monocytes (absolute), eosinophils, basophils (absolute), microscopic examination of blood and bone marrow smears.
- Coagulation parameters: activated partial thromboplastin time, prothrombin time, fibrinogen
- Clinical chemistry (serum): Albumin globulin ratio (calculated), alanine aminotransferase, albumin, alkaline phosphatase, aspartate aminotransferase, blood urea nitrogen, calcium, chloride, creatinine, creatine kinase, gamma-glutamyl trans-peptidase, globulin (calculated), glucose, high-density lipoprotein cholesterol, potassium, sodium, total bilirubin, total cholesterol, total protein, triglycerides

***For the 90-day dose repeat study only:***

- Urinalysis: volume, colour, appearance, glucose, bilirubin, ketone, blood, pH, protein, urobilinogen, nitrite, leukocyte, specific gravity

**Supplementary File 5.** **Anatomic pathology procedures in the 7-day dose range finding study**

Necropsy and gross examination of all organs was performed by a veterinary pathologist.

Organ weight collection was performed in all groups on the following organs: adrenal glands, brain, epididymides, heart, kidneys, liver, lungs, ovaries, pancreas, pituitary gland, prostate, seminal vesicles and coagulating glands, spleen, testes, thymus, thyroid and parathyroid glands, uterus with cervix. Histopathological evaluation was performed as follows:

- on the adrenal glands, brain, epididymides, heart, kidneys, liver, ovaries, pituitary gland, spleen, testes, thymus, thyroid and parathyroid glands, in the control (G1) and high dose groups (G5 and G9)
- on the thyroid and on all gross lesions, in all groups.

**Supplementary File 6.** **Anatomic pathology procedures in the 90-day dose range finding study**

Necropsy and gross examination of all organs was performed in all animals.

Organ weight collection was performed in all groups on the following organs: adrenal glands, brain, epididymides, heart, kidneys, liver, lungs, ovaries, pituitary gland, prostate, seminal vesicles and coagulating glands, spleen, testes, thymus, thyroid and parathyroid glands, uterus with cervix. Histopathological evaluation was performed on:

- all tissues of the animals from groups G1, G4 and G7: adrenal glands, aorta, biceps femoris muscles, brain, caecum, colon, diaphragm, duodenum, epididymides, eyes, femur bone with joint, Harderian glands, heart, ileum with Peyer’s patches, jejunum, kidneys, liver, lungs, mandibular and mesenteric lymph nodes, mammary gland, optic nerves, ovaries, oviducts, pancreas, pituitary gland, prostate, seminal vesicles and coagulating glands, rectum, salivary glands, sciatic nerve, skin, spinal cord, spleen, sternum, stomach, testes, thymus, thyroid and parathyroid glands, tongue, trachea, uterus with cervix, urinary bladder, vagina
- all gross lesions.
